# Unconventional Use of Balloon-Expandable Covered Stent in the Treatment of Iatrogenic Popliteal Pseudoaneurysm

**DOI:** 10.7759/cureus.41936

**Published:** 2023-07-15

**Authors:** Hamad J AlShatti, Ahmad Ameer, Hisham Rashid

**Affiliations:** 1 Department of Surgery, Vascular Surgery Division, Jaber Al-Ahmad Hospital, Kuwait City, KWT

**Keywords:** arterial stent, popliteal artery pseudoaneurysm, endovascular repair, covered stent, atrium v12

## Abstract

Popliteal artery pseudoaneurysm is a rare injury occurring in total knee replacement surgeries. The symptoms are usually pain and swelling on the affected side which prompts immediate investigations. Surgical and endovascular interventions are both available options for intervention with covered stents being the preferred choice. In this study, we report the case of a 72-year-old female diagnosed with right popliteal artery pseudoaneurysm following total knee replacement which was managed with the insertion of an Atrium Advanta V12 balloon-expandable covered stent (Atrium Medical Corp., Hudson, NH, USA).

## Introduction

As the superficial femoral artery descends through the adductor hiatus, the popliteal artery emerges as a continuation to it giving the posterior and anterior tibial arteries and laying against the posterior capsule of the knee at the knee joint [1,2]. The course and the location of the popliteal artery render it at risk of injury due to the mechanism and the dynamics of the knee joint [2-4]. Traumatic and iatrogenic causes are the main mechanisms behind pseudoaneurysms formation [1]. Popliteal pseudoaneurysm presents as an expanding pulsatile mass at the posterior aspect of the knee associated with pain mimicking deep venous thrombosis [1-4]. Both surgery and endovascular intervention are available options for treatment [1-3]. 

## Case presentation

A 72-year-old woman was admitted to the national orthopedics center in Kuwait for an elective bilateral total knee replacement on a background of degenerative joint disease.

**Figure 1:**
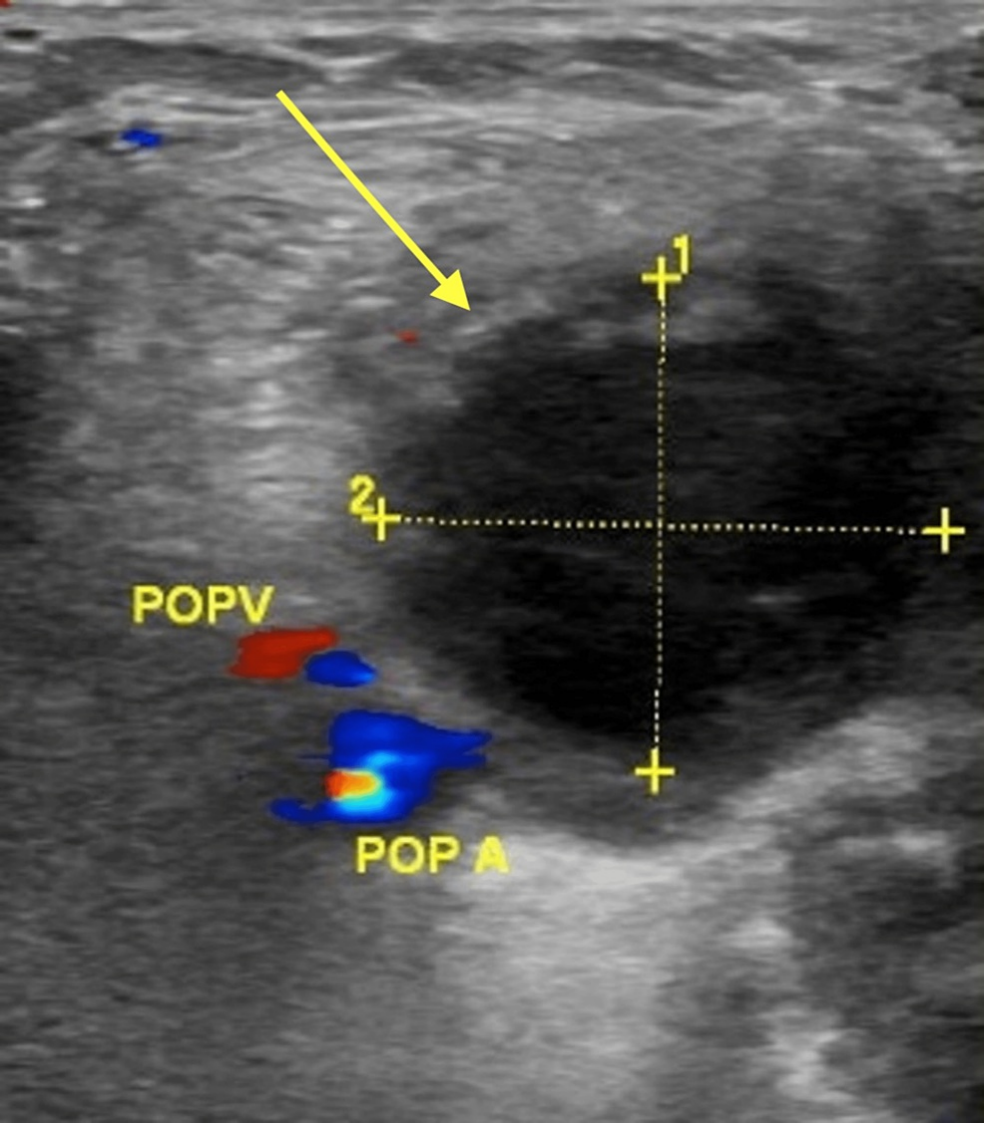
Duplex ultrasound of the right lower limb showing a 2.15 cm x 2.46 cm popliteal pseudoaneurysm (yellow arrow). POPV: popliteal vein, POPA: popliteal artery

A few days following the procedure, the patient complained of right calf pain, swelling and tightness. A duplex ultrasound was performed to rule out deep venous thrombosis and showed a right popliteal artery pseudoaneurysm (Figure 1). The patient refused to have Computed Tomography Angiography (CTA) due to concerns over the use of contrast.

Procedure

**Figure 2:**
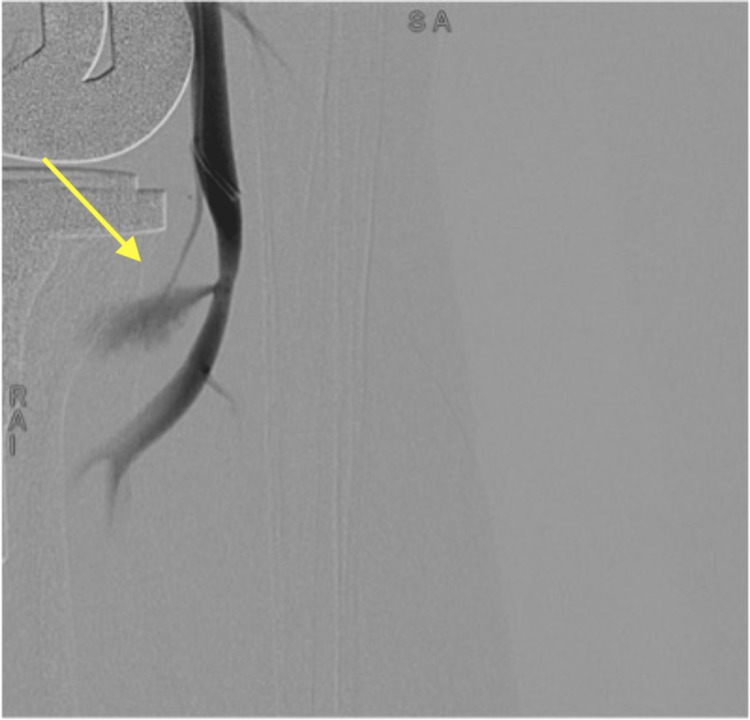
Arterial angiogram showing right popliteal pseudoaneurysm (yellow arrow).

A right antegrade femoral access was obtained, and 6 French femoral sheath introducer was used. Iodine contrast was injected using a 0.35 x 260 mm J TIP (Boston Scientific, Marlborough, MA, USA) which showed a large infrageniculate popliteal artery pseudoaneurysm (Figure 2).

**Figure 3:**
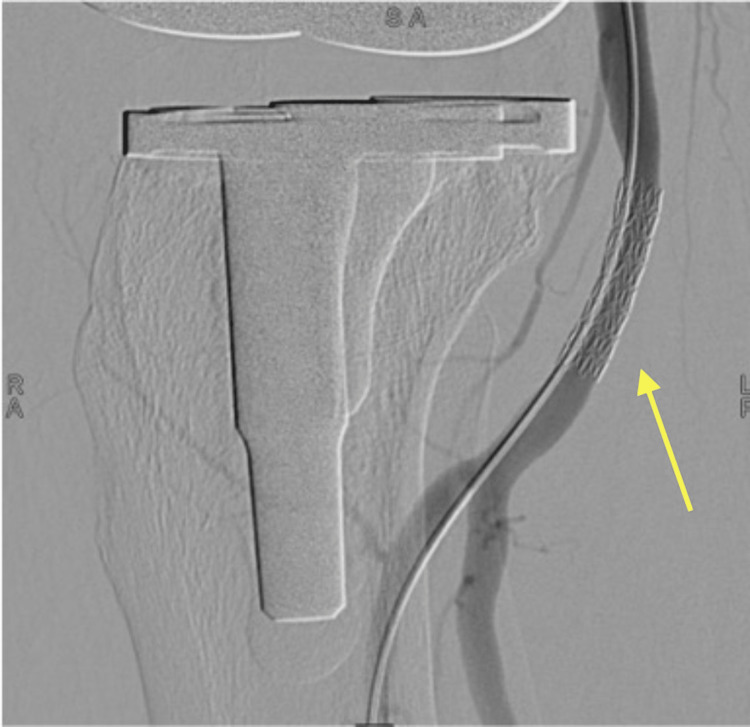
Completion angiogram showing the Atrium Advanta V12 balloon-expandable stent inserted with obliteration of the pseudoaneurysm (yellow arrow).

The knee joint was flexed and the angle of flexion was measured under fluoroscopy to avoid stent bending. A 5mm x 22 mm x 120 cm Atrium Advanta V12 covered stent (Atrium Medical Corp., Hudson, NH, USA) was inserted below the knee flexion point to cover the entry tear. Post-stenting imaging showed good vessel runoff and the obliteration of the pseudoaneurysm (Figure 3).

**Figure 4:**
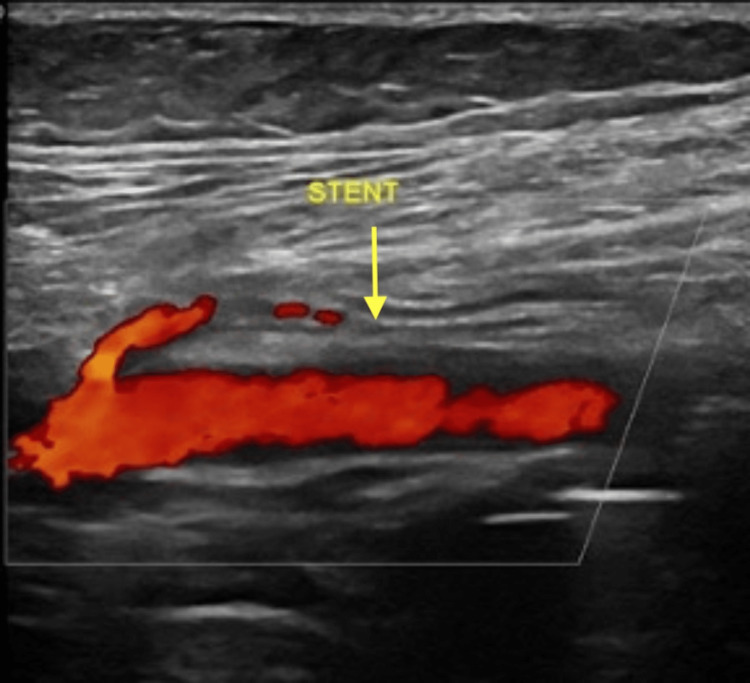
Follow-up duplex ultrasound of the right lower limb showing patent in-flow stent (yellow arrow).

The patient was discharged the next day on dual anti-platelet therapy and followed up regularly at the vascular outpatient clinic. During the follow-up, the right posterior tibial and dorsalis pedis pulses were palpable. A follow-up duplex ultrasound was done one year after the procedure which showed a patent right lower limb arterial tree from the right common femora artery to the right dorsalis pedis. Complete obliteration of the popliteal pseudoaneurysms with an in-stent flow site peak velocity of 44.5 cm/s, through stent peak systolic velocity of 48 cm/s, and distal to stent peak systolic velocity of 50 cm/s confirming stent patency (Figure 4).

It also showed a biphasic flow pattern over the posterior tibial, anterior tibial and dorsalis pedis arteries. Informed consent was obtained from all individual participants included in the study.

## Discussion

A pseudoaneurysm is a contained hematoma that is in direct connection with the artery due to a defect in the arterial wall [3,5]. In contrast to a true aneurysm, pseudoaneurysms do not involve the three layers of a vessel [3]. Total knee replacement is a common orthopedic intervention that is used as a treatment option for degenerative joint disease [2]. With an incidence rate of 0-3.5%, traumatic popliteal artery pseudoaneurysms following total knee replacement are generally rare [5]. The most common vascular complications encountered following total knee replacement were popliteal pseudoaneurysms (45%) followed by occlusion (33%) and transection (14%) [3].

According to Butt et al. (2010), popliteal artery pseudoaneurysms can occur during the release of severe flexion contractures and the traction of the artery leading to an intimal tear [4,6]. Another suggested mechanism is a direct puncture or laceration of the popliteal artery and its surrounding by any instrument used intraoperatively [2,4-7]. Schermer et al. (2021) proposed multiple theories leading to indirect trauma to the popliteal artery during total knee replacement. Tibial oscillating saw cut, placement of the retractor behind the tibial plateau, opening of the posterior capsule, or sacrificing the posterior cruciate ligament are all possible causes of intraoperative popliteal injury [3]. 

Common symptoms reported include the presence of an expanding pulsatile mass at the posterior aspect of the knee associated with pain, swelling, paraesthesia and an audible souffle [1-4,6,8]. If left untreated, serious complications can develop including acute limb ischemia, compartment syndrome and limb loss [1,3,6]. In this study, the patient presented with right calf swelling and pain.

The majority of popliteal pseudoaneurysms are diagnosed via duplex ultrasound due to the clinical suspicion of deep venous thrombosis. Duplex ultrasound is a safe, non-invasive and efficient diagnostic tool used to diagnose deep venous thrombosis initially and then in such cases, popliteal pseudoaneurysm [2,3,6]. CTA and magnetic resonance imaging (MRI) are other diagnostic tools that not only confirm the presence of a pseudoaneurysm but also show distal run offs and aid in pre-operative planning [2,3,6,7]. A reported disadvantage of CTA and MRI is the formation of artifacts in the presence of a prosthesis [3]. The patient in our case study had a duplex ultrasound which confirmed the diagnosis of a right popliteal artery pseudoaneurysm however the patient refused to have a CT angiogram.

Popliteal artery pseudoaneurysm can be treated in multiple ways. Ultrasound-guided compression therapy is used for small narrow-necked pseudoaneurysms [2,3]. Pseudoaneurysm resection with interpositional venous graft or arterial bypass is a common surgical intervention for popliteal diseases. This is due to the favorable anatomic location of the artery and the dynamics of the knee joint [1,3-11]. Nowadays, endovascular interventions have become more commonly used to treat such cases taking into consideration the presence of prosthesis, joint movement and the risk of graft bending and fracture with movement [1-3,6,7]. Owing to their flexibility and good durability for flexion during joint movement, Viabahn covered stents are widely used to exclude popliteal pseudoaneurysms [2,7,11]. 

In our study, we placed a balloon expandable stent at the entry tear. Balloon expandable stents are usually used in intracavity vessels where fear of bending, fracture and occlusion is minimal. The Atrium Advanta V12 balloon-expandable stent was placed below the flexion point which resulted in the immediate obliteration of the pseudoaneurysm and the patency of infrapopliteal vessels. The choice was made based on the lack of availability of the Viabahn covered stent and the refusal of the patient for further surgical intervention.

Endovascular interventions require prolonged anticoagulation and lifelong antiplatelet therapy. Our patient was kept on lifelong antiplatelet medication. Patients treated endovascularly need regular follow-up to ensure the patency of stents [2,4]. Our patient follows up regularly in the vascular outpatient clinic with duplex ultrasound. In her one-year follow-up, the patient had palpable posterior tibial and dorsal pedis arteries with the duplex ultrasound showing a patent stent with biphasic flow and complete obliteration of the pseudoaneurysm. 

## Conclusions

In conclusion, we report a case study of a patient diagnosed with iatrogenic popliteal artery pseudoaneurysm that was successfully treated with an Atrium Advanta V12 balloon-expandable covered stent. We believe that the use of balloon-expandable covered stents in short infrageniculate popliteal artery pseudoaneurysm can achieve satisfying results with complete obliteration of the pseudoaneurysm. Long-term patency can be achieved by making sure that the stent is placed at the correct location where it will not fracture or bend on knee flexion.
